# Bereavement help-seeking following an 'expected' death: a cross-sectional randomised face-to-face population survey

**DOI:** 10.1186/1472-684X-7-19

**Published:** 2008-12-14

**Authors:** David C Currow, Katrina Allen, John Plummer, Samar Aoun, Meg Hegarty, Amy P Abernethy

**Affiliations:** 1 Department of Palliative and Supportive Services, Flinders University, Sturt Road, Bedford Park, South Australia, 5042, Australia; 2 Flinders Centre for Clinical Change, Flinders University, Sturt Road, Bedford Park, South Australia, 5042, Australia; 3 Institute of Palliative and Supportive Care Research, Australia; 4 Southern Adelaide Palliative Services, Repatriation General Hospital, 700 Goodwood Road, Daw Park, South Australia, 5041, Australia; 5 Department of Anaesthesia, Flinders Medical Centre, Bedford Park, South Australia, Australia; 6 Western Australian Centre for Cancer and Palliative Care, Curtin University, Perth, Western Australia, Australia; 7 Division of Medical Oncology, Department of Medicine, Duke University Medical Centre, Durham, North Carolina, 27710, USA

## Abstract

**Background:**

This study examines the prevalence and nature of bereavement help-seeking among the population who experienced an "expected" death in the five years before their survey response. Such whole population data are not limited by identification through previous access to specific services nor practitioners.

**Methods:**

In a randomised, cross-sectional, state-wide population-based survey, 6034 people over two years completed face-to-face interviews in South Australia by trained interviewers using piloted questions (74.2% participation rate). Respondent demographics, type of grief help sought, and circumstantial characteristics were collected. Uni- and multi-variate logistic regression models were created.

**Results:**

One in three people (1965/6034) had experienced an 'expected' death of someone close to them in the last five years. Thirteen per cent sought help for their grief from one or more: friend/family members (10.7%); grief counselors (2.2%); spiritual advisers (1.9%); nurses/doctors (1.5%). Twenty five respondents (1.3%) had not sought, but would have valued help with their grief.

In multi-variate regression modeling, those who sought professional help (3.4% of the bereaved) had provided more intense care (OR 5.39; CI 1.94 to14.98; p < 0.001), identified that they were less able to 'move on' with their lives (OR 7.08; CI 2.49 to 20.13; p = 0.001) and were more likely not to be in full- or part-time work (OR 3.75; CI 2.31 – 11.82; p = 0.024; Nagelkerke's R^2 ^= 0.33).

**Conclusion:**

These data provide a whole-of-population baseline of bereavement help-seeking. The uniquely identified group who wished they had sought help is one where potentially significant health gains could be made as we seek to understand better any improved health outcomes as a result of involving bereavement services.

## Background

There are few baseline data to inform bereavement service planning for specialized palliative care/hospice services (SPCHS) where death may be 'expected'. In seeking to deliver more effective bereavement services as part of the work of SPCHS, it is useful to know the number and characteristics of people who already seek help for their grief, and the people from whom they access support currently [1].

Fundamentally, data that have underpinned bereavement planning models in palliative care have ignored the fact that only one in two people access palliative care services before an 'expected' death [2,3]. Such a model is blind to people where the deceased did not access SPCHS and hence cannot reflect the true rates of help seeking after an 'expected' death.

Existing literature on bereavement help-seeking in SPCHS is limited in terms of generalisability and applicability because it has not had a mechanism to contact bereaved survivors who did not have prior contact with services [4-7]. The denominator – the whole population who had experienced an 'expected' death – becomes the key to understanding what happens across the whole community [8]. These data are critical for bereavement service planning, especially as SPCHS work with increasing demands and relatively finite healthcare resources.

The aim of this study was to use a novel whole-of-population randomised survey to quantify the number of people who sought bereavement support, their characteristics and from whom they sought this help. The null hypothesis was that there would be no factors helping to identify people who sought help compared to those who did not after experiencing a recent 'expected' death of someone close to them.

## Methods

South Australia (SA) has an annual, random, face-to-face, cross-sectional health survey that approaches approximately 4500 people, the South Australian Health Omnibus, described in detail elsewhere [2,9-12]. On average more than 200 questions about health beliefs and behaviours (spanning smoking to childcare, respiratory disease to exercise habits) are included each year in interviews lasting between 60 and 90 minutes.

Selection of households to approach for interview sought to ensure statewide coverage. In metropolitan areas, a starting point was randomly selected for each of 375 Australian Bureau of Statistics metropolitan collector's district. In non-metropolitan areas, households were selected using 100 starting points state-wide. All towns with a population greater than 10,000 were included and towns above 1,000 were randomly included. In both metropolitan and non-metropolitan settings, 10 dwellings were randomly selected using a skip pattern of every fourth household. People living in communities of less than 1000 people, caregivers under the age of 15 and people in residential aged care facilities (nursing homes) were excluded from participating by this algorithm.

One interview per household was conducted with the person over the age of 15 who most recently had a birthday. Face-to-face interviews were conducted by trained interviewers. Data were anonymous and were double entered into the data base. Any missing responses were followed up by telephone. For quality assurance, 10% of each interviewer's respondents were randomly selected and re-contacted to confirm eligibility and responses. These processes apply to the whole survey, are unchanged since the survey's inception in 1991, and could not be modified. [2]

In the 2004 and 2005 (September – December) surveys, 14 broadly-based high level questions on palliative care issues were included of which seven directly related to bereavement [1]. Prompt cards were provided for selected answers to allow responses to be categorised [see Additional file 1].

The entrance question to the section on palliative and end-of-life care asked whether the respondent had experienced the death 'of someone close to them in the last five years from an illness such as cancer, emphysema or motor neurone disease?' If the person answered 'no' then no further questions from the palliative care section were asked, and the interviewer moved to the next topic area. Any respondents who had experienced an 'expected' death were asked if they had sought help for 'dealing with their grief and if so, from whom?'. An ability to 'move on' was a question used to incorporate concepts suggested in the grief literature more than one decade ago [13]. A sub-study sought responses based on suggestions from the pilot group as to what 'moving on' meant to people in the context of grief. [10]

Before use, all questions were piloted annually with 50 members of the general public for their detailed understanding. No changes were required as a result of feedback from the pilot.

### Ethics approval and consent

The survey was approved by a Department of Health Research Ethics Committee, and participants provided verbal consent to participate.

### Analyses

Data were directly standardized against the whole state (2001) for gender, 10 year age group, socio-economic status, and region of residence (urban, suburban, outer metropolitan, regional, rural and remote). Descriptive statistics were used to summarize respondent characteristics and frequency of responses.

Relationships between categorical variables were assessed using chi squared and regression analyses for continuous variables. Variables explored in univariate analyses included: characteristics of the deceased (diagnosis, time since death, comfort in the last two weeks of life); demographic data of the respondent (gender, age, country of birth, highest level of education, current work status, marital status, pre-tax household income, rural/metropolitan place of residence); caregiving characteristics (relationship to the deceased, intensity of care and period of time for which care was provided, caregivers' expectations between the time of diagnosis and death, and the ability to 'move on' with their life); and service issues (SPCHS use).

Logistic regression models were created to identify the strongest predictors of people who reached out for any bereavement support and for professional bereavement support. From univariate analyses, items were included in the multivariate analyses if they had a p < 0.10.

## Results

Of the 9500 buildings approached, 307 (3.2%) were vacant, could not be accessed or were businesses, and contact could not be made after six visits with a further 1064 (11.2%). Having made contact, reasons for not participating included: too busy/not interested; (1819, 19.1%), illness or mental incapacity (133; 1.4%), and language barriers (142, 1.5%). One person terminated the interview while in progress. Having made contact with 8129 households, 6034 people completed interviews (participation rate – 73.3% (unweighted data)) [see Additional file 2].

### General characteristics of the bereaved

All data reported from this point are from population weighted data. One thousand nine hundred and sixty five respondents (31.9%) had experienced the death of "someone close to them" from an expected death in the preceding five years. The average age of people who were bereaved was 45.3 years (range 15–92; standard deviation 17.7) and 48.5% were male. Fifteen per cent were close relatives of the deceased (spouse/son/daughter/parent). The deceased had a cancer diagnosis in 82.0% of cases with the most frequently encountered other causes of expected death including emphysema/lung disease (9.6%); neuro-degenerative diseases (3.4%) and end-stage heart failure (3.3%).

### Seeking help after bereavement

The majority of the bereaved (1667; 84.8%) did not identify that they had sought help. Respondents identified reaching out to one or more of: family and friends 210 (10.7%); spiritual adviser 38 (1.9%); grief counselor 43 (2.2%) and doctor or nurse 29 (1.5%) for support.

Basic characteristics of the deceased, the bereaved and service use are compared to a person's access of bereavement support (all support including family and friends, and professionals only), [see Additional file 3] and age [see Additional file 4].

Twenty five people (19 women, 6 men) identified that they had not had help with the grief but would have valued such input. Nine were in a current relationship. Sixteen people in this group were under the age of 45, and only one person was born in a country where English was not the first language. Twenty people were on incomes of less then AU$60,000 per year with missing data for three people. With ten missing responses, only 4 people were participating in full or part time work. Eighteen had completed high school or less. For 18 respondents, the person had been dead for more than one year.

Using univariate analyses, the group who reached out for help were more likely to be female (18.4% of females versus 9.4% of males; p < 0.001), report that the period between diagnosis and death as 'worse than expected' (19.3% for 'worse' or 'far worse' versus 'far better', 'better' or 'as expected' 10.1%; p < 0.001), report that they were unable to 'move on' with their lives (47.3% not able to 'move on' with their lives had sought help from bereavement services compared to 11.3% of people who were able to 'move on' with their lives; p < 0.001), had provided higher levels of caregiving (day-to-day or intermittent hands-on care 30.7% reach out for help compared with 9.5% of people who provided rare or no hands-on care) for the deceased (p < 0.001) and were currently less likely to be participating in the workforce (17.4% who were not working full- or part-time sought help with grief compared with 8.8% of people in full- ot part-time work; p < 0.001).

Significant factors were incorporated into a logistic regression model for predicting use of any bereavement service (Nagelkerke's R^2 ^0.217). Factors included in the model which were significant contributors to people seeking help with grief include people who were unable to 'move on' (OR 4.88; CI 2.72 to 8.77; p < 0.001), providing day-to-day or intermittent hands on care (OR 2.25; CI 1.38 to 3.68; p = 0.001), female gender (OR 1.95; CI 1.21 to 3.12; p = 0.006), and people not in full or part time work (OR 1.78; CI 1.12 to 2.83; p = 0.016). Factors found not to be significant include caregiver expectations between diagnosis and death, whether the deceased was a spouse, time since death, metropolitan/rural place of residence, income, and age.

In multivariate regression models to predict characteristics of the 68 (3.4% of all bereaved) people in the sub-group who reached out for professional help (where this includes counselors, doctors, nurses and spiritual advisers), three factors were significant: an inability to 'move on' with their lives (OR 7.08; CI 2.49 to 20.13; p < 0.001); higher levels of care (defined by a period of day-to-day or intermittent hands on care) that they provided (OR 5.39; CI 1.94 to14.98; p = 0.001) and not participating in the full- or part-time workforce (OR 3.75; CI 2.31 – 11.82; p = 0.024). Nagelkerke's R^2 ^rose to 0.33 in this model. Factors in the model that were not significant included gender, caregiver expectations for the time between diagnosis and death, age, spousal relationship and use of a palliative care service.

### 'Moving on'

The bereaved population conceived the three most important aspects of 'moving on' to incorporate: a sense that life was 'getting back to normal' (54%); 'accepting death as part of life (34%); and an ability to 'stop dwelling on the past' (17%).

## Discussion

One criticism of bereavement research by Forte is a lack of a "targeted, well-defined patient population"[14]. As key work in grief and bereavement progresses [15-17], this current study helps to define better a group of people who self-identify as reaching out for bereavement support after a death which was 'expected' in their life. Despite relatively small numbers of people reaching out for services of professionals, statistically significant predictors of help seeking were found. Such findings bring focus to the question of what ideal bereavement support should look like.

Who should access systematized bereavement services and when should they be offered? Is it sufficient for people to reach out for help themselves or should services identify and follow people at higher risk of complicated grief? Is what is currently offered by SPCHS really specialist bereavement services or simply a 'bereavement approach' to people after they have experienced an expected death? These findings may open the way for more detailed empirical work to define the net clinical and social benefits that could be derived from properly structured and evaluated bereavement services for people currently not accessing services or not 'moving on' with their lives. Specifically, when these results are read in the context of a small but identifiable cohort of people who perceive that they do not 'move on' with their lives after an expected death, the real challenge for systematized bereavement services (in contrast to individual bereavement counselors) becomes clear [10]. How do we build systems to better meet complex needs not identified until years after a life-changing event such as the death of someone close? [18,19]

Importantly, the study identifies a group of people who have not accessed services, but believe that this could have been of benefit to them. Most case series are based around people who have sought help or people who are likely to be bereaved in the foreseeable future as identified through case lists from clinical services [20,21]. The population of people not seeking help from bereavement services has been difficult to identify and hence poorly studied until now. Studies to date have failed to capture the whole target population because of the systematic exclusion of potential respondents. The group thus omitted is of particular concern to planners of bereavement services as they are currently not receiving any support. By contrast, the study also highlights that the majority of people deal with bereavement without explicit family or professional help [22-24].

### What other literature do these data support?

Of the data available in the literature, bereavement help-seeking [25] in Utah saw 11.5% of respondents seek professional help for their grief [25] but the study had a low response rate. Connor studied bereavement help seeking in a population of users of hospice care and found 16% subsequently used professional services [26]. An Australian study [27] reported bereavement help seeking as it relates to culture and religion. In that study 3.3% of people sought psychiatric or psychological help and a much higher number (23%) sought medical or pharmaceutical help for bereavement. Health service utilization in this setting is a complex relationship that may not reflect need. [28]

Why do the studies in the literature have such widely varying rates of professional help seeking for bereavement? The difference is most likely the denominator. The current study approached a representative random sample of the population older than 15 years of age. The other studies have focused on contacting people who have already been identified by their use of health services.

### Levels of accessing professional support and unmet need

The population numbers of people needing professional help reflect proposed models of bereavement support services [28]. Even adding together those who sought help, and those who perceive that they would benefit from professional help would increase to only 6% of all the bereaved those people would access professional help with their grief after experiencing a recent 'expected' death of someone close. This is a 40% increase over current levels of help sought from a professional.

### Factors found to be predictive of professional help seeking for bereavement

'Moving on' is a consistent predictor of help seeking. The results build on the original concept by Prigerson and her group and helps validate the concept in her screening tool for complicated grief [13]. Given that complicated grief requires the passing of time before the diagnosis can be made [18,19,29-32], this current study also explored whether there was any pattern in the timing in which help was sought. We did not identify a shift to professional help as time passed, but the passing of years after the death before concerns of abnormal grief can be diagnosed makes identifying and supporting the bereaved a challenge for health services.

Caregiving has long been identified as a specific risk factor for complicated grief [23,33]. People in closer relationships are more likely to experience poor grief outcomes [34,35] and may therefore be more likely to seek help. It is not unexpected that being a more involved caregiver also is positively associated with seeking professional help.

### Work status

The respondents' current work status was a predictor for any help or professional help with bereavement. Intuitively, it is not surprising that lower levels of workforce participation are seen in people whose complexity of need has been such that they have reached out for help. The personal and social implications of lower rates of workforce participation in this group need to be further explored.

### Gender

Several studies have already found women are more likely than men to discuss ongoing grief [36].

### 'Expected death'

Even in the setting of a diagnosed life-limiting illness, it is of note that one in five people in the same data set did not access SPCHS because death was 'unexpected' [37] a recognised risk factors for complicated grief [20,38,39]. The fact that death in the palliative setting can be 'unexpected' means that the identification that someone is 'palliative' should not equate with a presumption that their relatives or friends automatically 'expect' death. The diagnosis of a life-limiting illness may not forewarn loved ones about impending death [40].

### Generalisability

This cross-sectional, patterns-of-care study is not limited by self-selection nor gate-keeping by family or professionals – common research challenges in bereavement. The patterns of service uptake are likely to reflect the care for communities with similar socio-demographics, and social health systems. The age range in this study reflects the universality of expected death across the age range and is not limited to the elderly alone.

In the Omnibus data, there is no representation of people from communities of less than 1000 people including remote farming and mining communities. People from an Aboriginal or Torres Strait Islander background and people whose place of birth was not an English-speaking country are also potentially under-represented in the population approached. Although representative of the adult population across most of the life-span, the Omnibus data do not explore bereavement from sudden deaths: perinatal mortality; suicide; motor vehicle nor industrial accidents; war nor medical causes for sudden death including acute myocardial infarction. As children and young adolescents are not interviewed, their experiences of 'expected death' are not reflected in the findings. The omission of residential aged care facilities from case finding algorithms will under-estimate the impact of grief on an elderly population who are most likely to have experienced expected deaths of people close to them.

### Limitations – methods

Given the nature of the face-to-face interview about palliative and end-of-life care embedded within a much larger health survey, it has not been possible to ask questions about pre-existing or simultaneous psychopathology, nor draw any conclusions on any cause-and-effect health consequences of grief. Structured interviews such as Omnibus are not the ideal way to elicit complex diagnostic issues about depression, anxiety or other co-existing psychopathologies.

The Omnibus results rely on identification that an encounter was 'seeking help'. People may, for example, seek help for a somatic symptom that is based in bereavement rather than a physical problem. Even accepting that this may happen, the Omnibus data are still an accurate reflection of those who can identify that the help that they have sought and received was for their bereavement.

Any survey that seeks to reflect on patterns of health service use is limited by service availability, people's knowledge of these services and potential clients' expectations of health care. Publicly funded bereavement services in South Australia are limited with heavy reliance on volunteers to complement a small group of health professionals from a range of clinical backgrounds.

The factors explored are necessarily high level questions given the nature of the survey. Other than the demographic questions, this study used non-validated questions, and any findings are association only, rather than implying cause and effect.

In people seeking professional help, the regression model developed only accounts for a fraction of the variance. This suggests that more detailed work needs to be done to understand fully the factors that predict uptake of professional bereavement support after an expected death. Methods other than population surveys are likely to be able to add detail to this regression model.

### Limitation – sample

Apart from the populations not surveyed outlined in Methods, one other significant and unavoidable omission will be caregivers who themselves have died between relinquishing the caregiving role and the survey being performed [23].

## Conclusion

### Impact on Policy and Practice

This study is the first step in better understanding what is happening across the whole population as people experience the consequences of an 'expected' death. The need to identify the people who have not accessed adequate support is an important target for service planners. Although the number of people who did not seek help but believe they would have provided benefit was small, it may also be that this is the cohort where the greatest health gains can be made by providing more comprehensive bereavement care [41]. The level of unmet need suggested in these results should help to influence more formal planning for professional bereavement services.

### Implications for research

Having established this baseline level of professional and non-professional bereavement support sought at a whole-of-population level, there is need to better understand the characteristics of the people who do not access adequate support. What is the level of day-to-day consequences these people experience? [42] Ultimately, are there ways of helping people to identify their need to reach out for help in a timely way? [13,19]

Lack of participation in the workforce in the long-term has enormous social and financial consequences for a person. Further work needs to explore any patterns to changed workforce participation while in the caregiving role and, more importantly, having relinquished the role at the time the person dies.

This findings of this study now open the way to explore the relationship between grief, depression and other psychopathologies at a population level rather than only people accessing clinical services [22,36] and a mechanism to correlate bereavement outcomes with social supports, and coping skills [43]. Such research will need to utilise a population-based methodology for engaging participants beyond the broadly based Health Omnibus methodology and questions.

## Competing interests

The authors declare that they have no competing interests.

## Authors' contributions

DCC and APA were responsible for the conceptualization and refining research ideas: DC, AA, KA, MH carried out the literature search: DCC, APA, JP, KA created the research design: DCC, APA were responsible for the instrument selection, construction and design: DCC, APA, KA, JP, SA were involved in data analysis: All authors were involved in preparing and reviewing the manuscript.

## Funding

The authors wish to thank the Daw House Hospice Foundation for their generous provision of discretionary funds to help support this research.

## Pre-publication history

The pre-publication history for this paper can be accessed here:



## Supplementary Material

Additional file 1 **In the 2004 and 2005 (September – December) surveys, 14 broadly-based high level questions on palliative care issues were included of which seven directly related to bereavement**. Prompt cards were provided for selected answers to allow responses to be categorised. Click here for file

Additional file 2 **Having made contact with 8129 households, 6034 people completed interviews (participation rate – 73.3% (unweighted data))**. Click here for file

Additional file 3 **Basic characteristics of the deceased, the bereaved and service use are compared to a person's access of bereavement support (all support including family and friends, and professionals only)**. Click here for file

Additional file 4 **Basic characteristics of the deceased, the bereaved and service use are compared to a person's access of bereavement support (all support including family and friends, and professionals only) and age**. Click here for file
