# Supplementary material for: Bereavement help-seeking following an 'expected' death: a cross-sectional randomised face-to-face population survey
Source: BMC Palliat Care. 2008 Dec 14;7:19. doi: 10.1186/1472-684X-7-19 (PMC2637838; doi:10.1186/1472-684X-7-19)
Supplement: Additional file 1 — In the 2004 and 2005 (September – December) surveys, 14 broadly-based high level questions on palliative care issues were included of which seven directly related to bereavement. Prompt cards were provided for selected answers to allow responses to be categorised. [file 1472-684X-7-19-S1.doc]

**Additional file 1**

**2004,2005 South Australian Health Omnibus bereavement-related survey questions.** (Bulleted responses were on prompt cards for easier coding)

1. In the past five years, has anyone close to you died of a terminal illness like cancer, motor neurone disease or emphysema?

If yes, could you please say what their main illness was?

1. What was your relationship to this person?

- Spouse/partner
- Parent
- Child
- Sibling
- Other relative
- Friend
- Other(specify)

1. Think back to the period from the time that the person was first diagnosed with their illness until their death. What was your most involved level of care during this person’s illness?

- Day-to-day hands on care
- Intermittent hands on care
- Rare hands on care
- Didn’t provide any care but they were still close to me

1. Again, think back to when they were first diagnosed. Was the period after their diagnosis through to and including their death better or worse than you would have expected?

- Better than expected
- As expected
- Worse than expected
- I didn’t know what to expect

1. Since this person died, have you sought help to deal with your grief?

- No I have not needed to seek help to deal with my grief
- No but I would have liked some help to deal with my grief
- Yes, from a trusted friend or family member
- Yes, from a grief counselor
- Yes from a spiritual advisor
- Yes, from a nurse or a doctor
- Yes, from some other person

6. Since this person died, have you been able to move on with your life?

- I have been able to move on with my life
- I am starting to move on with my life
- I have not been able to move on with my life

7. Did this person who died of a terminal illness use a palliative care service?
