# Supplementary material for: Bereavement help-seeking following an 'expected' death: a cross-sectional randomised face-to-face population survey
Source: BMC Palliat Care. 2008 Dec 14;7:19. doi: 10.1186/1472-684X-7-19 (PMC2637838; doi:10.1186/1472-684X-7-19)
Supplement: Additional file 2 — Having made contact with 8129 households, 6034 people completed interviews (participation rate – 73.3% (unweighted data)). [file 1472-684X-7-19-S2.doc]

**Additional file 2**

**Participant flow - South Australian Health Omnibus 2004, 2005**

(Unweighted data). **Participation rate 74.2% (6034/8129).**

**9500 households randomly approached across South Australia**

Approach to household

Vacant houses, holiday homes, businesses

n = 307 (3.2%)

Unable to contact after 6 visits (different times of day/evening, days of the week)

n = 841 (8.6%)

Problems making contact leaving 8129 (86.6%) households with whom contact was made.

Unable to gain access to building

n = 84 (0.9%)

Potential respondent away for the duration of the survey

n = 139 (1.5%)

Declined to participate (not interested, too busy)

n = 1819 (19.1%)

Illness, mental incapacity

n = 133 (1.4%)

Unable to speak English

n = 142 (1.5%)

Difficulties encountered having made contact with the household

**Completed interviews**

**n = 6034** (63.5%)

Terminated interview

n = 1

Interviews

Number of people who had someone close to them die from and expected illness in the last 5 years

n = 1965 (20.7%)

Interviewees who had experienced an ‘expected death’ and hence could answer questions on bereavement. This formed the data set that was weighted to the standard (whole) population
