# Supplementary material for: Bereavement help-seeking following an 'expected' death: a cross-sectional randomised face-to-face population survey
Source: BMC Palliat Care. 2008 Dec 14;7:19. doi: 10.1186/1472-684X-7-19 (PMC2637838; doi:10.1186/1472-684X-7-19)
Supplement: Additional file 3 — Basic characteristics of the deceased, the bereaved and service use are compared to a person's access of bereavement support (all support including family and friends, and professionals only). [file 1472-684X-7-19-S3.doc]

**Additional file 3**

**Help sought across the community after an expected death of someone close. 2004, 2005 South Australian Health Omnibus Survey – weighted data**.

|  | **Any help sought for bereavement** | | | | **Professional help sought for bereavement*** | | | |
| --- | --- | --- | --- | --- | --- | --- | --- | --- |
| **Factor (n)** | **n (%)** | **p value** | **OR** | **CI** | **n (%)** | **P value** | **OR** | **CI** |
| **Characteristics of the deceased and their death** | | | | | | | | |
| **Life-limiting illness** | | | | | | | | |
| Cancer (1596) | 222 (13.9) | 0.777 | 0.954 | 0.690-1.319 | 52  (3.3) | 0.587 | 0.847 | 0.464-1.545 |
| Non-cancer (366) | 53 (14.5) |  |  |  | 14  (3.8) |  |  |  |
| **Comfortable or very comfortable in the last two weeks of life** | | | | | | | | |
| Yes (282) | 36 (12.8) | 0.716 | 1.079 | 0.715-1.629 | 11  (3.93) | 0.204 | 1.624 | 0.763-3.456 |
| No (779) | 93  (11.9) |  |  |  | 19 (2.4) |  |  |  |
| **Time since death** | | | | | | | | |
| ≤1 year (793) | 97 (12.2) | 0.063 | 0.777 | 0.595-1.014 | 20 (2.5) | 0.067 | 0.611 | 0.359-1.039 |
| > I year (1157) | 176 (15.2) |  |  |  | 47 (4.1) |  |  |  |
| **Respondent issues** | | | | | | | | |
| **Gender** | | | | | | | | |
| Male (954) | 90 (9.4) | 0.000 | 0.463 | 0.353-0.605 | 23  (2.4) | 0.014 | 0.531 | 0.319-0.884 |
| Female (1012) | 186 (18.4) | ‘ |  |  | 45 (4.4) |  |  |  |
| **Country of birth** | | | | | | | | |
| Aust/UK/NZ (1805) | 246 (13.6) | 0.074 | 0.684 | 0.450-1.040 | 61 (3.4) | 0.509 | 0.764 | 0.344-1.700 |
| Other (160) | 30 (18.8) |  |  |  | 7  (4.4) |  |  |  |
| **Highest level of education** | | | | | | | | |
| ≤ high school (887) | 130 (14.7) | 0.475 | 1.097 | 0.850-1.416 | 36  (4.1) | 0.187 | 1.384 | 0.852-2.247 |
| > high school (1079) | 146  (13.5) |  |  |  | 32 (3.0) |  |  |  |
| **Current workforce participation #** | | | | | | | | |
| Full or part time (635) | 56 (8.8) | 0.000 | 0.460 | 0.317-0.667 | 10 (1.6) | 0.003 | 0.325 | 0.150-0.701 |
| Other (430) | 74 (17.4) |  |  |  | 20 (4.7) |  |  |  |
| **Place of residence #** | | | | | | | | |
| Metropolitan (1290) | 197  (15.3) | 0.025  *** | 1.377 | 1.040-1.823 | 45  (3.5) | 0.790 | 1.073 | 0.639-1.802 |
| Non-metropolitan (674) | 78  (11.6) |  |  |  | 22  (3.3) |  |  |  |
| **Relationship to the deceased** | | | | | | | | |
| Spouse (61) | 29  (47.5) | 0.000 | 6.080 | 3.615-10.226 | 15  (24.6) | 0.000 | 11.388 | 5.984-21.675 |
| Other (1904) | 247 (13.0) |  |  |  | 53 (2.8) |  |  |  |
| **Level of care** | | | | | | | | |
| Day-to-day or intermittent care (420) | 129  (30.7) | 0.000 | 4.216 | 3.225-5.512 | 44 (10.5) | 0.000 | 7.744 | 4.619-12.983 |
| Other (1545) | 147 (9.5) |  |  |  | 23 (1.5) |  |  |  |
| **Length of care provided (n=464; caregivers only)** | | | | | | | | |
| ≤ I year (371) | 96 (25.9) | 0.861 | 1.035 | 0.705-1.519 | 31  (8.3) | 0.510 | 1.218 | 0.680-2.175 |
| > I year (93) | 56  (26.5) |  |  |  | 21 (10.0) |  |  |  |
| **Expectations of the time from diagnosis until death**^^ | | | | | | | | |
| Better than or as expected (969) | 98 (10.1) | 0.000 | 0.470 | 0.354-0.622 | 18  (1.9) | 0.000 | 0.384 | 0.204-0.650 |
| Worse than expected (688) | 133 (19.3) |  |  |  | 34 (4.9) |  |  |  |
| **Ability to ‘move on’ with life** | | | | | | | | |
| Able to move on (1818) | 205 (11.3) | 0.000 | 0.141 | 0.097-0.205 | 40  (2.2) | 0.000 | 0.110 | 0.057-0.213 |
| Not able to move on (132) | 62 (47.3) |  |  |  | 26 (19.7) |  |  |  |
| **Household Issues** | | | | | | | | |
| **Income (232 people chose not to give a response) #** | | | | | | | | |
| ≤AU$60K p.a. (1010) | 164 (16.2) | 0.008  *** | 1.457 | 1.100-1.931 | 45  (4.5) | 0.012  *** | 2.066 | 1.157-3.684 |
| >AU$60K p.a. (724) | 85 (11.7) |  |  |  | 16  (2.2) |  |  |  |
| **Service issues** | | | | | | | | |
| **Palliative care service use (318 respondents did not know)** | | | | | | | | |
| Yes (1014) | 168 (16.6) | 0.172 | 1.214 | 0.919-1.604 | 48 (4.7) | 0.057 | 1.698 | 0.978-2.946 |
| No (633) | 89 (14.1) |  |  |  | 18 (2.8) |  |  |  |

* ‘Professional’ includes counsellors, doctors, nurses and spiritual advisers

** Excludes 240 respondents who ‘did not know what to expect’

*** Not significant using the Boneferroni correction 0.05/15 = 0.003 for multiple Chi-squared assessments from same data set

# Factors that potentially change if the bereaved person was living in the same household as the deceased
