# Supplementary material for: Bereavement help-seeking following an 'expected' death: a cross-sectional randomised face-to-face population survey
Source: BMC Palliat Care. 2008 Dec 14;7:19. doi: 10.1186/1472-684X-7-19 (PMC2637838; doi:10.1186/1472-684X-7-19)
Supplement: Additional file 4 — Basic characteristics of the deceased, the bereaved and service use are compared to a person's access of bereavement support (all support including family and friends, and professionals only) and age. [file 1472-684X-7-19-S4.doc]

**Additional file 4**

**Regression analysis for help seeking after an ‘expected’ bereavement. 2004,2005 South Australian Health Omnibus Survey (weighted data).**

| Any help sought for grief | N (%) | Mean age | Standard deviation | range | P value |
| --- | --- | --- | --- | --- | --- |
| yes | 276 (14.0) | 45.58 | 17.85 | 15-90 | 0.784 |
| no | 1689 (86.0) | 45.27 | 17.68 | 15-92 |  |
| Professional help sought for grief |  |  |  |  |  |
| yes | 68 (3.5) | 49.90 | 17.02 | 16-85 | 0.030 |
| no | 1898 (96.5) | 45.15 | 17.71 | 15-92 |  |
